# Perspectives of pregnant and postpartum women and obstetric providers to promote healthy lifestyle in pregnancy and after delivery: a qualitative in-depth interview study

**DOI:** 10.1186/s12905-020-0896-x

**Published:** 2020-03-04

**Authors:** Manasa S. Ayyala, Janelle W. Coughlin, Lindsay Martin, Janice Henderson, Nneamaka Ezekwe, Jeanne M. Clark, Lawrence J. Appel, Wendy L. Bennett

**Affiliations:** 1 Rutgers New Jersey Medical School, Department of Medicine, Division of General Internal Medicine, 185 South Orange Avenue, MSB B624, Newark, NJ 07103 USA; 2 Department of Psychiatry and Behavioral Sciences, The Johns Hopkins University School of Medicine, 5510 Nathan Shock Drive, Suite 1100, Baltimore, MD 21224 USA; 3 Department of Medicine, Division of General Internal Medicine, The Johns Hopkins University School of Medicine, 2024 E. Monument St, Suite 2-616, Baltimore, MD 21205 USA; 4 Department of Gynecology and Obstetrics, The Johns Hopkins University School of Medicine, 600 N. Wolfe St, Nelson Building, Baltimore, MD 21287 USA; 5 School of Medicine, University of Mississippi Medical Center, Jackson, MS 39216 USA

**Keywords:** Qualitative research, Gestational weight gain, Healthy lifestyle, Health behavior, Pregnancy, Postpartum

## Abstract

**Background:**

Pregnancy provides an opportunity to promote healthy lifestyle behaviors. This study’s aim was to explore the perspectives of pregnant and postpartum women and obstetric providers around behavioral lifestyle changes in pregnancy and postpartum.

**Methods:**

We conducted a qualitative study with pregnant and postpartum patients recruited from 2 prenatal care clinics at an urban, academic hospital in the United States. In-depth interviews with 23 pregnant or postpartum women and 11 obstetric providers were completed between October 2015–April 2016. Interviews were audio-recorded and transcribed verbatim. We coded transcripts for thematic content and applied the PRECEDE-PROCEED framework for results to directly inform program development.

**Results:**

Six themes highlighted the predisposing, enabling and reinforcing factors that enable and sustain health behavior changes in pregnancy and postpartum: 1) “Motivation to have a healthy baby” during pregnancy and to “have my body back” after delivery, 2) Pre-pregnancy knowledge and experiences about pregnancy and the postpartum period, 3) Prioritizing wellness during pregnancy and postpartum, 4) The power of social support, 5) Accountability, 6) Integration with technology to reinforce behavior change.

**Conclusions:**

In this qualitative study, pregnant and postpartum women and obstetric providers described themes that are aimed at encouraging lifestyle changes to promote healthy weight gain in pregnancy and can directly inform the development of a behavioral weight management intervention for pregnant and postpartum women that is patient-centered and tailored to their needs.

## Background

Excessive weight gain during pregnancy is linked to adverse maternal and perinatal outcomes including increased rates of cesarean section [1], preeclampsia, gestational diabetes and childhood obesity [2, 3]. In 2009 the National Academy of Medicine (formerly Institute of Medicine) revised its guidelines on healthy weight gain during pregnancy based on pre-pregnancy body mass index (BMI) [4]. Despite these recommendations, there has been a steady increase in weight gain in pregnancy, with 73% of pregnant women gaining weight above the recommended ranges in all pre-pregnancy BMI categories [5].

Pregnant women have unique barriers to healthy weight management and positive lifestyle changes [6], including physical changes due to pregnancy, lack of knowledge about weight gain, exercise and dietary guideines during pregnancy, and difficulty with time management due to family, work, and health care-related demands [7]. Despite these barriers, pregnancy and prenatal care provide a unique opportunity to capitalize on a woman’s motivation to have a healthy baby and make behavioral changes aimed at healthy weight management [8]. Growing evidence supports the efficacy of behavioral interventions in limiting gestational weight gain, thereby reducing pregnancy complications, as well as future maternal weight gain and obesity [9–13]. Additionally, behavioral interventions aimed at weight loss in the postpartum period can help women lose retained weight and reduce future risk of obesity [14]. However, commercially-available weight loss programs like *Weight Watchers,* exclude pregnant women [15], and patient-centered interventions that are accessible for diverse patient populations, integrated into prenatal care settings and have the potential for scalability are lacking. Understanding factors that motivate women to make and sustain behavioral changes in pregnancy and to sustain them postpartum is needed to inform the design and tailoring of weight management programs for pregnant and postpartum women.

The primary aim of this qualitative study was to explore the perspectives of both pregnant and postpartum women and obstetric providers around behavioral changes in pregnancy, specifically focused on healthy lifestyle in pregnancy and in the postpartum period that promotes healthy pregnancy weight gain and postpartum weight loss. In addition, we sought to understand barriers, needs and preferences of pregnant and postpartum patients and their obstetric providers around developing a behavioral intervention that could become part of routine prenatal care. In order to most effectively and efficiently translate our findings into informing intervention development, we applied the PRECEDE-PROCEED framework [16], designed for program planning and development,.

## Methods

### Study design and sample selection

This study was conducted in accord with prevailing ethical principles and approved by the Institutional Review Board at The Johns Hopkins University School of Medicine.

Between October 2015 and April 2016, purposive sampling was used to recruit pregnant and postpartum women from two prenatal clinics that serve a diverse patient population at The Johns Hopkins Hospital. Obstetric providers were recruited through email invitations targeting both community and academic, mid-level and attending providers.

Pregnant and postpartum women were eligible for inclusion into the study if they were age 18 or older, receiving obstetric care at The Johns Hopkins Hospital or Johns Hopkins Bayview Medical Center, English speaking, currently pregnant or had delivered her baby within the past 3–12 months, and had a pre-preganancy BMI > 20. Women who had a known diagnosis of pre-pregnancy type 1 or type 2 diabetes mellitus were excluded. Thirty women were screened and deemed eligible. Following their prenatal visits their obstetric providers assessed their interest in hearing more about the study and offered a flyer. If she was interested, the interviewer further introduced the study and scheduled the interview, either immediately following the visit or for a mutually convenient future time.

Obstetric providers were eligible if they cared for pregnant or postpartum women. Resident physicians were excluded from this study.

### Data collection

Baseline sociodemographic information was collected from both pregnant and postpartum women as well as providers based on a self-report questionnaire administered at the time of study consent.

Two trained interviewers, one a dietician and the other a physician (M.S.A), conducted the interviews with pregnant and postpartum women. One physician investigator (W.L.B) conducted all provider interviews. Interviews with pregnant and postpartum women as well as with providers lasted on average, 1 hour in duration. Interviews were conducted until thematic saturation was achieved in data analysis using an iterative process [17]. Interviews were conducted in-person or by phone, based on participant preference and were audiotaped and transcribed verbatim. Two different semi-structured interviewer guides were created for the interviews with pregnant and postpartum women and with obstetric providers. The interview guide for pregnant and postpartum women started with open-ended questions focused on participants’ knowledge and health goals and then moved to more structured questions regarding their individual experiences with lifestyle modifications (Additional file 1). Providers were asked questions regarding their experiences with counseling pregnant and postpartum women regarding healthy weight gain in pregnancy, and additional questions to assess their opinion on implementing a behavioral counseling intervention that would use health coaching into clinical care (Additional file 2).

### Data analysis

Data were analyzed in two stages with researchers analyzing interviews with pregnant and postpartum women followed by interviews with providers, given the different interview guides and goals for these two groups (Additional file 1 and Additional file 2). For each stage, two investigators sequentially coded transcripts for thematic content using editing style analysis [18].A final coding template with descriptive and conceptual codes was created after each stage, through an iterative process guided by the PRECEDE-PROCEED health programming framework [16]. We selected the PRECEDE-PROCEED framework because of its ecological approach to the identification of priorities to help inform the creation of an effective population health program. Key in this framework is identifying those factors which should be of highest priority and thus become the focus of an intervention, by identifying predisposing, enabling, and reinforcing factors that influence behavior change. Emerging themes were grouped within these factors. The coding process was thus inductive and deductive and the coding template was revised to incorporate feedback from research team members. After the coding guide was finalized, all transcripts were coded using Atlas.ti (Version 1.0.49, Atlas.ti Scientific Software Development GmbH, Berlin).

## Results

**Fig. 1:**
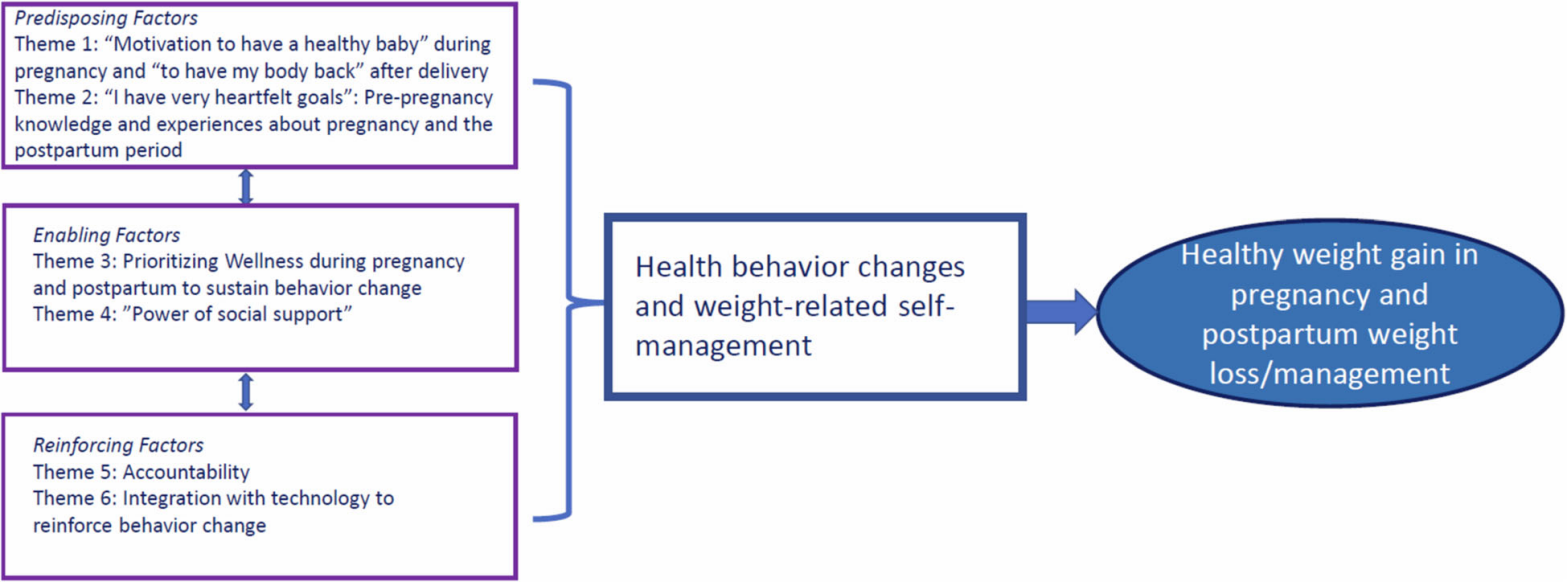
Six key themes in the PRECEDE-PROCEED model (14) that influence behavior change in pregnancy and postpartum

Of the 30 pregnant and postpartum women screened for participations, 23 women elected to complete their interview (7 declined). All eleven providers offered an interview participated. Tables 1 and 2 describes participant characterisitcs. Figure 1 shows the application of the PRECEDE-PROCEED framework to the 6 themes identified within the categories of predisposing (facilitate or hinder motivation for change), enabling (make possible a desired change) and reinforcing (influence continuation of the behavior) factors that can influence behavior change. Below we describe each of the themes aligned with these factors with representative quotes from pregnant or postpartum women and obstetric providers.

**Table 1:** Characteristics of 23 pregnant and postpartum women who participated in interviews

Patients, *N* = 23	*N* (%)
Age (years)	
< 25	3 (13)
25–30	10 (44)
31–35	6 (26)
> 35	4 (17)
Pregnant	14 (64)
Race	
White	10 (44)
Asian/Pacific Islander	4 (17)
Black	7 (30)
Other	2 (9)
Years of Education	
< 13	6 (26)
14–17	5 (22)
> 17	12 (52)
Owns a smart phone	20 (87)

**Table 2:** Characteristics of 11 obstetric providers who participated in interviews

Providers, *N* = 11	*N* (%)
Age < 40	3 (27)
Female	9 (82)
Race	
White	9 (82)
Black	1 (9)
Other	1 (9)
Specialty	
General OB/GYN	3 (27)
Maternal Fetal Medicine	2 (18)
Nurse Midwife or NP/PA	5 (45)
Health Coach	1 (9)

### Predisposing factors: motivation and pre-pregnancy knowledge/experience (themes 1–2)

#### Predisposing theme #1: motivation

The first predisposing theme was women’s level of motivation for making behavior changes in pregnancy and postpartum. In pregnancy, women were motivated by wanting to have a healthy baby and delivery and, in the postpartum period, women were motivated by wanting “my body back.” One pregnant woman stated:

*… I just want to make sure that I have a healthy delivery...and the baby will just be health [y] – both of us will be healthy...And, of course take care of him. You know – all throughout his life, and you know... [I] have to be healthy first so I can take care of him.” (Pregnant woman).*

In addition to motivation for having a healthy baby, women were motivated to have an uncomplicated labor and delivery and for many, to successfully breastfeed their infant. Obstetric providers also considered women’s motivation to have a healthy baby as an opportunity for counseling about behavior changes. One nurse midwife, illustrated this opportunity to engage pregnant women in the following way:

*You’re catching them at a time when … they know they’re in this pregnancy for a limited amount of time, you know? So it’s not [as] overwhelming … we’re initially just asking them … to focus on being healthy just for this [finite] amount of time. (Certified Nurse Midwife)*

During the postpartum period, women described their motivation to make healthy lifestyle changes to improve their body image and “feel like me again.” One postpartum woman said,

*I mean pregnancy just kind of … not destroys your body, but it makes you feel like you are a different person … I guess exercising is more for me to feel like me again, and to feel happy with the way that I look and the way that I feel. (Postpartum woman)*

Another postpartum woman illustrated a desire for wanting her clothing to feel similar to how it felt before her pregnancy as follows:

*It would it would be nice to have the ten pounds off and be back to sort of fitting into things a little bit better. I think that it’ll be better for my body. I could fit in more clothing which … it would be nice because that’s definitely like an ‘ugh’ feeling when you like, try to put something on and you are like, ‘Oh yeah, that doesn’t fit anymore. (Postpartum woman)*

#### Predisposing theme #2: pre-pregnancy knowledge and experience

The second predisposing theme was women’s pre-pregnancy knowledge and experiences about importance of healthy behaviors, including eating well and physical activity in pregnancy and postpartum. Although many pregnant and postpartum women expressed basic knowledge about the importance of eating well and being physical active, they had different opinions on what they should and should not do to achieve a healthier lifestyle. One pregnant woman described the benefits of physical activity in pregnancy in the following way:

*I wanted to bounce back quickly and – you know, if I go [to] the gym maybe labor will be a little easier, my body will be conditioned to that sort of thing, so – (Pregnant woman)*

Some women described having health goals like taking prenatal vitamins during pregnancy and breastfeeding after pregnancy, because of past knowledge and experiences with previous pregnancies. In the postpartum period, women commented on their lack of preexisting knowledge about the challenges of breastfeeding their infants, and expressed a desire to have had more information during pregnancy, and support in the postpartum period:

*[Before I had my baby] I was under the impression that if you tried [to breastfeed your infant] and you just sat there [then] you did it. And [if] you just breastfed all day, it would work. But it just didn’t. (Postpartum woman)*

Obstetric providers described examples of women who already had knowledge and skills about the importance of physical activity and nutrition noting that these women were able to continue a healthy lifestyle. For example, one obstetric provider stated:

*I think the patients that come in already at a normal healthy weight and that have good healthy behaviors already are the ones who are more likely to ask specific questions, like can I keep running? Can I keep going to yoga? I had somebody recently who was asking me about weight lifting in, like getting into the third trimester, and those are usually the people who are already doing these things and they want to be able to continue. (Obstetrician)*

### Enabling factors: Priorizing wellness and social support (themes 3–4)

#### Enabling theme #3: prioritizing wellness

Pregnant and postpartum women described the importance of overall “wellness” as enabling them to make and maintain behavior changes. Wellness was defined broadly and beyond their pregnancy-related health, including mental/emotional health, sleep quality, feeling in control of their own time and reducing stressors.

In particular, lack of quality sleep was a major barrier to dietary and physical activity changes, especially postpartum when women had newborns. One postpartum woman described lack of sleep as a barrier to exercise as illustrated by the following quote:

*Between four and six months [my baby] was waking up like every hour like every night. It was just … it was really bad. It just made me feel so tired during the day that the idea of moving, getting up and doing things was really not [feasible] … So I feel like that’s been a big problem. (Postpartum woman)*

Some women shared their struggles with emotional changes during and after pregnancy, even postpartum depression. One postpartum woman described her mood in the following way:

*I don’t think I had full scale postpartum depression with either of my pregnancies, but I definitely had the baby blues pretty badly especially with the first one … . Thinking back to it … ugh … I was not in a good mood for the first bunch of months, I think my husband noticed it more, but I’m not sure he necessarily wanted to like drag me to a doctor (Postpartum woman)*

Another postpartum woman described her lack of energy in pregnancy as, “[W] hen I come home I’m just like – I don’t want to do anything. I don’t [want to talk with anyone] … my [low] energy level kind of sometimes puts a strain on [me].” A postpartum woman described how stress can be a trigger for her to eat high calorie foods:

*I end up eating [or] drinking a soda or having some candy or something, because I'm like oh I’m felling stressed [and] this would make me feel better. (Postpartum woman)*

Obstetric providers noted the importance of addressing women’s wellness in pregnancy, including mood and sleep, and not just focusing on the patients’ weight. One certified nurse midwife said, “I think it’s important not just to focus on the number [her weight] but just being healthy in general.*”*

#### Enabling theme #4: social support

The majority of participants shared stories highlighting the importance of strong social support from family and friends, to enable them to make and sustain health behavior changes in pregnancy. One pregnant woman highlighted the benefit of having peer support from another pregnant friend:

*Sometimes you need another pregnant women’s point of view so they can say, “I know what you’re going through. (Pregnant woman)*

Social support from friends and peers with similar experiences was especially important in the postpartum period:

*I just had a baby two weeks ago. [My family member] was like oh, really-- Look at your cheeks, look at your belly. I felt like I wasn’t doing [well] with my weight. When I read about [and saw other women’s] experiences, I knew I wasn’t alone in this. Actually, I was doing [well]. (Postpartum woman)*

Obstetric providers also commented on their role as providing support for their patients through behavioral changes:

*[W]omen want to get a little pampered and feel like, you know, they’re being taken care of and you want to make sure that she’s feeling okay and that she’s doing okay and that she’s getting what she needs and, you know, just basically a little extra attention um, from a healthcare provider I think can do a lot … (Certified Nurse Midwife)*

### Reinforcing factors: accountability and technology integration (themes 5–6)

#### Reinforcing theme #5: accountability

Participants described the importance of knowing someone outside of their immediate family and friend network to be an “accountability partner” who could provide positive reinforcement to help them stay on track with reaching health goals in pregnancy and postpartum. For most women, their healthcare providers served this role. One participant stated,

*You know so the one thing is accountability I would eat more, exercise less [if] no one else [was] looking. . . (Postpartum woman)*

Another woman described,

*[I like] feeling like you have some sort of support, you know, whether you needed it or not, but to know that someone’s checking in on you and they really care about how you’re doing and the baby and trying to make your life easier*. *(Postpartum woman)*

One obstetric provider highlighted the importance of positive reinforcement and said, “It might be something you just say, [like] ‘hey, you’re doing great with the weight … Keep up the good work.’”

#### Reinforcing theme #6: technology integration

Participants were specifically asked about how their clinics could support their efforts in achieving healthy lifestyles in pregnancy. In particular, participants discussed their use of technology, including mobile phones and mobile applications, which could facilitate their behavior change and enable communication with providers between visits. One woman remarked “I would use [it] everyday” with regard to the ability to interface with her moble phone to help with behavior change. Another woman remarked that her health care providers would “know where I’m coming from and they can work with me better” noting this would “also help them on their end.”

Obstetric providers discussed the importance of integrating future clinical programs on healthy lifestyle within the electronic medical record to facilitate their ability to review patients’ progress. One obstetric provider discussed this point in the following way:

*I’m a bigger fan of an [electronic] referral [to a healthy lifestyle program] just because … then there’s tracking of it … . Reading other providers’ notes or reading the [behavioral counselor, i.e. health] coaches notes … that’s something you can eventually weave in … to your other visits and things like that … . And then too, … [to] read what the patients’ … .responses [are] or if there’s trends or repeating issues you can touch base [about these isues] in [the] visit, you know? [In real time]. (Certified Nurse Midwife)*

## Discussion

In this study involving one-on-one interviews with pregnant and postpartum patients and obstetric provides, we identified six themes within the PRECEDE-PROCEED model that could inform the development of a prenatal care program that promotes healthy lifestyle in pregnancy and postpartum: 1) “Motivation to have a healthy baby” during pregnancy and to “have my body back” after delivery, 2) pre-pregnancy knowledge and experiences about pregnancy and the postpartum period, 3) Prioritizing wellness during pregnancy and postpartum, 4) The power of social support, 5) Accountability, and 6) Integration with technology to reinforce behavior change.

Prior studies have similarly underscored the influence of psychosocial factors (i.e. women’s motivations for change, body image, social support) on health behavior change in pregnancy [19–21]. Addressing these psychosocial factors in lifestyle programs and clinical care can improve weight gain outcomes in pregnancy [22]. Our study supports other research that women are motivated to make healthy lifestyle changes during and after pregnancy [23]. Our findings also underscore the importance of leveraging women’s desires to have a healthy pregnancy outcome and also improve their postpartum body image to enhance their motivation for health behavior change during pregnancy and after [19, 22, 24, 25]. A recent systematic review found that inaccurate body image perceptions and reduced knowledge about healthy weight gain were significantly associated with excessive gestational weight gain [25]. These findings suggest the need for psychoeducational and cognitive approaches to be included within behavioral interventions in pregnancy to address women’s health expectations, body image, and misperceptions.

In this study, pregnant and postpartum women highlighted the importance of addressing factors associated with their wellness other than weight, eating, and physical activity, to enable behavior change during and after pregnancy. Poor sleep can contribute to reduced physical activity and poor dietary behaviors (e.g., snacking, preference for calorically dense foods) [26], and previous work has shown that sleep is an important mediator for health behavior change in pregnancy [27]. Chang and colleagues [26] conducted focus groups in low-income overweight and obese women to identify factors that influence barriers to physical activity and eating behaviors. Their participants discussed their stressful life events and psychosocial stressors (e.g., poor communication with significant others), which negatively impacted their ability to manage their weight; in fact, women expressed a desire to learn more about stress management and problem solving skills, which could be incorporated into future programs. The Moms in Motion Program [28] was a 16-week community-based weight gain prevention program for low-income, overweight and obese mothers who were 6 weeks to 4.5 years postpartum. In a qualitative analysis, the authors shared that their intervention may have overlooked the importance of addressing negative thoughts and depression, particularly since they are strongly associated with everyday stressors, which may even intensify during pregnancy and postpartum (e.g., worry about baby’s health, childcare worries, financial concerns, etc.). Although a systematic review conducted by Kapadia and colleagues on identifying psychological factors associated with gestational weight gain [25] did not report an association between depression, anxiety and stress, and excessive gestational weight gain, addressing these wellness components may be important for engaging and retaining participants, especially lower income women. Future research should explore these factors as potential mediators of dietary and physical activity changes.

Similar to weight loss interventions in non-pregnant adults, our study highlighted the crucial role of social support and accountability during pregnancy and postpartum to promote and sustain health behavior changes [29]. Our participants noted the unique challenges of the postpartum period, a time when many women are lost to follow-up from research, as well as health care, and thus, a time period with missed opportunities for behavioral support and feedback [30]. Interventions based on social cognitive theory, which focus on increasing and eliciting social support, may be effective in pregnant and postpartum women, as suggested in a review of studies aimed at examining postpartum heath behaviors in women with gestational diabetes [31].

With improving provider and patient access to mobile technologies, we found that both pregnant and postpartum women and their providers desired greater technology features in weight management programs [32, 33]. Electronic health and mobile technologies (e.g. electronic health records, web-based and mobile-accessible platforms, text messaging) can enable health behavior change and improve adherence over scheduled in-person weight management interventions [30]. In fact, remotely-delivered behavioral health coaching has been shown to be an effective tool in the management of a wide range of health conditions, including obesity [34], and is often a service provided by health plans [35]. Growing evidence supports remote delivery of behavioral interventions as efficient and potentially more accessible for young women [36, 37], particularly in the postpartum period when attrition rates are highest [38].

Several limitations to this qualitative study should be considered. First, because we recruited women who were already receiving prenatal care, our results are limited to women who have already overcome the barrier of access to care and thus, our sample is less representative of women who are not in or delay their care. Second, our study population was recruited from one academic institution, which may limit the generalizability of our results to community settings. Third, reflexivity of researchers may have contributed to bias as serving in the position of health care professionals may have changed the approach to the study as well as participants’ approach to how they answered interview questions with a possible leaning toward socially acceptable answers with regard to specific questions about health care behaviors.

The themes we identified have implications for the development of a patient-centered intervention focused on healthy weight gain and lifestyle during pregnancy and postpartum. Our results not only highlight barriers and facilitators to healthy weight gain and other lifestyle changes, but they also emphasize the critical importance of incorporating wellness and social support components into behavioral interventions for pregnant and postpartum women.

## Conclusions

In conclusion, our study explored pregnant and postpartum women and obstetric providers’ perspectives about behavior change during and immediately following pregnancy. We identified six key themes that could directly inform the development of a behavioral intervention aimed at encouraging healthy lifestyle changes for healthy weight gain in pregnancy and weight loss postpartum.

## Supplementary information

**Additional file 1.** Pregnant/postpartum participant interview guide

**Additional file 2.** Provider Interview Guide

## Data Availability

The datasets generated and/or analyzed during the current study are not publicly available due to containing identifiable information for participants (i.e. interview transcripts), which are not possible to de-identify, but are available from the corresponding author on reasonable request.

## Abbreviation

BMI: Body mass index
