# Supplementary material for: Perspectives of pregnant and postpartum women and obstetric providers to promote healthy lifestyle in pregnancy and after delivery: a qualitative in-depth interview study
Source: BMC Womens Health. 2020 Mar 4;20:44. doi: 10.1186/s12905-020-0896-x (PMC7057562; doi:10.1186/s12905-020-0896-x)
Supplement: Supplementary file 1 — Additional file 1. Pregnant/postpartum participant interview guide [file 12905_2020_896_MOESM1_ESM.pdf]

Supplement #1

Patient Interview Guide

<u>Describe study purpose:</u>

Thank you for your time and participation in this interview. It should last about 1 hour.

The goal of the interview is to gather your feedback about a program we have been creating to help women have a healthier lifestyle in pregnancy and after delivery. What we mean by "lifestyle" is eating healthy foods, limiting fast food, increasing how much exercise you get, and keeping you from gaining too much weight.

This interview will have a few parts. First, I'd like to hear about how your pregnancy is going / (if postpartum) how your pregnancy went and how things are at home with the new baby. Next, I'm going to share with you information about the pregnancy program that we're developing for people to use online, either with a computer or on your smartphone. Last, we're going to do an in-depth look at one of the sections of the program that is supposed to teach you about how to have a healthy lifestyle in pregnancy.

Let's begin with a few questions about your pregnancy (and postpartum period, if applicable).

<u>Part One: Questions about Healthy Lifestyle and Health Goals</u>

1. Let's talk about your health during your pregnancy/delivery/postpartum. What were some of your questions and concerns?
   - Healthy diet?
   - Exercise?
   - Weight gain?
   - Stress management?
   - Sleep?
   - Mood?
   - Other?

2. Tell me about…What areas of your life have been/were the most stressful during your pregnancy/delivery/postpartum?
   - Work?
   - Family/Friends?
   - Finances?
   - Mental/Physical health?
   - Time?
   - Other?

3. Tell me about…How you cope with stress during your pregnancy/delivery/postpartum?
   - Take a walk / do something physical

- Watch TV / listen to music / distract yourself
- Eat/drink/smoke etc.
- Deep breaths / relaxation
- Talk to someone

4. Tell me about…Who in your life has been supportive throughout your pregnancy/delivery/postpartum period? What makes this person supportive? Can you share examples of how this person has been supportive? (or not been)
    - Spouse/partner
    - Friend
    - Family member
    - Therapist/counselor
    - A member of my faith community
    - Other
    - Website/apps

5. Tell me about…What kinds of social support you feel are the most important to have as you're experiencing pregnancy/delivery/postpartum?
    - Spouse/partner
    - Friend
    - Family member
    - Therapist/counselor
    - A member of my faith community
    - Online community
    - Other

6. Tell me about your eating in pregnancy…When you think about "healthy eating," what does that mean to you? How does it make you feel? Is it something that is important to you? Tell us about what you eat. and if not, what makes it hard to do.
    - Prompts on barriers and facilitators to healthy eating.

7. Tell me about your physical activity in pregnancy…When you hear "exercise," what does mean to you? How does it make you feel? Is it something that is important to you? Tell us about whether you exercise and if not, what makes it hard to do.
    - Is it boring / difficult / stressful / not interesting?
    - Is it exciting / easy / enjoyable / interesting?
    - Something you want? Don't want?
    - Something you wish you had more time for?
    - Something you don't care about one way or the other?

8. If you had an opportunity to talk to someone regularly about your lifestyle while pregnant, to whom would you prefer to talk?
    - A friend/relative who has been pregnant before
    - Someone who is currently pregnant
    - A parent

- Someone who works in healthcare
- A doctor/midwife
- A health coach
- Other

5. *Describe the development of our health coaching program.* Would you be open to talking to someone like a counselor or coach about your lifestyle (diet, exercise, stress, sleep) while pregnant? Why or why not?
