# Supplementary material for: Perspectives of pregnant and postpartum women and obstetric providers to promote healthy lifestyle in pregnancy and after delivery: a qualitative in-depth interview study
Source: BMC Womens Health. 2020 Mar 4;20:44. doi: 10.1186/s12905-020-0896-x (PMC7057562; doi:10.1186/s12905-020-0896-x)
Supplement: Supplementary file 2 — Additional file 2. Provider Interview Guide [file 12905_2020_896_MOESM2_ESM.pdf]

Supplement #2

Provider Interview Guide

<u>Describe study purpose:</u>
Thank you for your time and participation in this interview. It should last about 1 hour.

The goal of the interview is to gather your feedback about a health coaching program in pregnancy and after delivery to help women of all pre-pregnancy BMI's manage their weight and reach healthy lifestyle goals.
During the interview I will ask you some questions about what might be helpful for you and your patients in a health coaching program with an online component during and after pregnancy.

Q1. Many providers find it difficult and discouraging to discuss weight gain in pregnancy with their patients, especially with overweight or obese women.

Could you describe a typical visit with a pregnant patient, when you address how much weight to gain during pregnancy and how to have a healthy diet and be physically active.

PROMPTS

a. What do you usually discuss with your patients related to weight gain during pregnancy? (e.g. assess motivation, physical activity, diet, medications)

b. What guidelines or resources do you use to make your recommendations (e.g. IOM recommendation, ACOG)?

c. What type of dietary approach, if any, do you tend to recommend to your patients (e.g., Weight Watchers, DASH, My Plate method, calorie range, increase fruits and veggies, etc.) *Be sure to ask in an open-ended way before giving some of these prompts.*

d. What resources do your recommend to patients (e.g. websites, handouts)?

e. Do you refer patients somewhere or to use any programs? If so, when? And what/where? Any app or website recommendations?

f. What are some of the challenges you face in having patients follow through with your recommendations?

g. How do you advise patients to manage or lose weight after delivery?

h. How do you advise women about weight loss who are breastfeeding?

i. Would you like a health coaching program available to all women, regardless of whether they had pre-pregnancy overweight or obesity? Why or why not?

j. What risk factors do you think would exclude patients from being in a standardized weight management program? (e.g., multiple pregnancies, high risk (age, comorbidities, etc.)

Q2. Think about your patients who have struggles with their weight, weight gain in pregnancy and losing weight after delivery:

PROMPTS

a. What were some of their biggest challenges? What challenges did you experience the most in offering services to them?
b. What tools or resources do you think could have helped them even more?

Q3. Can you think of a patient or several patients who managed weight gain successfully… what helped her / them to be successful?

PROMPTS

a. What was your role?
b. Role of other providers?
c. Social support?
d. Programs?
e. Did this patient have any challenges she was able to overcome, and how?
f. What do you think could help more patients be successful with managing weight gain?

Now we're going to talk about the health coaching program we're developing for pregnant and postpartum women. In this program, women would be assigned a health coach. The health coach will use behavioral techniques and a patient-centered approach called motivational interviewing to help them understand healthy lifestyle behaviors during and after pregnancy and to figure out the best ways to reach these goals. Coaching occurs by telephone at a mutually convenient time, for about 20 minutes per call, likely weekly. We'd love your feedback about making this program work for your patients.

Q4. What are some key features of the program you think we'd need to make it successful for you and your patients?

PROMPTS

a. Do you have any prior experience referring patients to a health coaching program? If so, please explain. What did you like? What did you not like?
b. How often during pregnancy do you think women should be in touch with their health coaches (weekly, every 2 weeks)?
    a. How about postpartum?

Q5. Some online programs have women track their weight daily and also their diet to calculate calories. We are considering having women track weight and also specific health behaviors like intake of fast food and sugary drinks and steps or minutes of physical activity.

a. What are your thoughts about whether your patients will be able to track specific behaviors throughout pregnancy and postpartum? What about calories? What behaviors do you think are most important to have patients track?

Q6. Do you have any tips about what might keep patients engaged in the program throughout pregnancy and during the year after delivery?

Q7. Are there any pregnancy resources or programs that you would want a weight management program "linked with", such as fetal growth or "what to expect" types of guides?

Q5. How would you envision your role to be like in a health coaching program being offered to your patients?

PROMPTS

a. If providers will have the ability to refer patients into the program, how would this work in your practice? (e.g. epic referrals) What would have to change in your current work flow to make it work?
b. What do you think are the key features we would need in the program to make it work for you and your patients? What would you want your role to be after referring patients?
c. Can you foresee any barriers to implementing the program we would want to consider in advance, such as problems with getting reports into the epic?

We are developing weight and diet / activity progress reports that we will make available to providers when their patients are in the program. Here is an example of progress reports we used in another health coaching study. We'd like your feedback on how to adapt these to be useful for you.

Q5. What are some key pieces of information you'd like to see in the report?

a. How often would you want to be able to see reports?
b. How would you want to access/receive them?
c. How could you envision using a progress report in a visit with a patient?

Thank you for your time. Is there anything you think we missed?
